# Estimating Risk to Responders Exposed to Avian Influenza A H5 and H7 Viruses in Poultry, United States, 2014–2017

**DOI:** 10.3201/eid2505.181253

**Published:** 2019-05

**Authors:** Sonja J. Olsen, Jane A. Rooney, Lenee Blanton, Melissa A. Rolfes, Deborah I. Nelson, Thomas M. Gomez, Steven A. Karli, Susan C. Trock, Alicia M. Fry

**Affiliations:** Centers for Disease Control and Prevention, Atlanta, Georgia, USA (S.J. Olsen, L. Blanton, M.A. Rolfes, S.C. Trock, A.M. Fry);; US Department of Agriculture, Riverdale, Maryland, USA (J.A. Rooney, D.I. Nelson, T.M. Gomez, S.A. Karli)

**Keywords:** influenza, avian influenza, influenza virus, viruses, H5 subtype, H7 subtype, influenza in birds, poultry, disease outbreak, risk, exposure, responders, respiratory infections, personal protective equipment, zoonoses, influenza risk assessment tool, United States

## Abstract

In the United States, outbreaks of avian influenza H5 and H7 virus infections in poultry have raised concern about the risk for infections in humans. We reviewed the data collected during 2014–2017 and found no human infections among 4,555 exposed responders who were wearing protection.

In late 2014 and early 2015, highly pathogenic avian influenza (HPAI) A(H5N2), A(H5N1), and A(H5N8) viruses were detected in poultry and wild birds in the United States and Canada. A fully Eurasian A/goose/Guangdong/1/1996-lineage (gs/GD/96) HPAI H5N8 clade 2.3.4.4 virus was detected along with reassortants of gs/GD/96 H5N8 with North American wild bird lineage low pathogenicity avian influenza (LPAI) viruses (reassortants H5N2 and H5N1). The gs/GD/96 H5N8 virus was detected sporadically along the Pacific flyway with few detections of the reassortant H5N2 virus in poultry. However, shortly after the reassortant H5N2 virus was detected in the Midwest, it rapidly spread, infected domestic poultry flocks in 15 states, and required a massive response effort to depopulate >50 million birds (*1*). Although ample data regarding potential public health impacts were available for the gs/GD/96 H5N1 virus, less was known about the more recent subclades (H5 2.3.4.4). Since 2003, gs/GD/96 had been reported to have caused 860 human infections in 16 countries (*2*). Furthermore, reported illness from the gs/GD/96 H5 virus infections has been severe; this virus caused deaths in 53% of persons infected (*3*).

On the basis of the theoretical risk for transmission from poultry to humans, in 2015 the Centers for Disease Control and Prevention (CDC) and the US Department of Agriculture (USDA) Animal and Plant Health Inspection Service (APHIS) drafted monitoring recommendations for persons potentially exposed to low pathogenicity and highly pathogenic H5 and H7 viruses as a part of the official USDA APHIS response efforts in the United States (Table 1). The recommendations were phased in during late 2015 and called for active monitoring for illness in persons exposed to virus through these response activities (e.g., handling infected birds or carcasses or working in a virus-contaminated environment) during and for 10 days after the last exposure. We reviewed the data and proposed a revision to the recommendations for monitoring.

**Table 1:** Information on monitoring guidelines for persons responding to an outbreak of avian influenza in poultry, United States, 2014–2017*

Area of information	Guidance
Definition of active monitoring	Active monitoring indicates that someone contacted each responder daily to assess responder health status. Monitoring for signs of illness was recommended for the duration of the exposure and for 10 d after the last exposure.
Responders asked to report if they had new onset or worsening of any of the following signs and symptoms	Fever or feeling feverish/chills; cough; sore throat; runny or stuffy nose; eye tearing, redness, irritation (pink eye); sneezing; difficulty breathing; shortness of breath; fatigue (feeling tired); muscle or body aches; headaches; nausea; vomiting; diarrhea; seizures; rash
Specimen	Respiratory or conjunctival
Who monitored	
Mobilized responders	USDA/APHIS safety officers or contractor safety officers performed daily monitoring on-site
Demobilized responders	State or local health department officials made contact with demobilized responders at least twice, upon arrival and at the end of the 10-d period
Who performs testing	State health department
Who is tested	Decision based on recommendations of state health department after assessing clinical illness, exposure, and use/breach of personal protective equipment

*USDA/APHIS, US Department of Agriculture Animal and Plant Health Inspection Service.

## The Study

The objective of this evaluation was to estimate the risk for infection in persons responding to outbreaks by using data retrospectively obtained during December 2014–September 2017. We used several sources of data. We identified the domestic poultry detection events that were reported to USDA. Then, to identify the number of persons exposed during a response (i.e., responders), we used USDA reports from each incident and limited to persons who were deployed to the field. For the numerator, we used state reports to CDC of any ill responders and testing results. Specimens were tested for influenza viruses by reverse transcription PCR. We calculated the percent positive among exposed official responders and estimated 95% binomial CIs.

We found 264 detections of H5 or H7 viruses in poultry across 20 states; most (≈92%) were during the outbreak of infection with gs/GD H5 HPAI virus during 2014–2015, and 4,555 responders were potentially exposed to a virus (Table 2). Responders were from 3 main groups: USDA, USDA contractors, and state/local agriculture. All responders were recommended to receive seasonal influenza vaccine and were assumed to have properly worn adequate personal protective equipment (PPE) (*4*); no data were systematically collected on PPE breaches. CDC did not recommend antiviral chemoprophylaxis for persons using proper PPE. The APHIS Health and Safety and PPE Guidance for HPAI and APHIS-CDC monitoring guidance applied to all responders. Twenty-three persons became ill and were tested; no human infections with avian influenza viruses were detected. The risk for infection with avian influenza for responders was low, although our power to make this statement with confidence varied by year and virus because there was a wide range (74–3,962) of number of persons involved in each response. The data were most robust during the outbreaks of infection with gs/GD/96 H5N8 and reassortant HPAI H5N2 viruses in poultry during 2014–2015.

**Table 2:** Influenza virus detection in poultry and persons potentially exposed, ill and tested, United States, 2014–2017*

Time	No. states	Virus	No. premises	No. domestic poultry	Total responders potentially exposed (by affiliation)	No. ill persons positive for avian influenza/no. tested (95% CI)	Other pathogens detected
Dec 2014–Jun 2015	15	H5N2, H5N8, H5N1†	242: all HPAI	50.4 million	3,962 (3,009 contractors, 773 USDA; 180 state/local)	0/5 (0–0.001)	Not systematically collected
Jan 2016‡	1	H7N8	9: 1 HPAI, 8 LPAI	414,000	519§ (374 contractors; 78 USDA; 67 state/local)	0/16¶ (0–0.007)	1 coronavirus OC43, 1 rhino/enterovirus
Mar 2017	4	H7N9	13: 2 HPAI, 11 LPAI	272,000	74# (45 USDA; 29 state/local)	0/2** (0–0.001)	1 coronavirus, 1 influenza B virus

*HPAI, highly pathogenic avian influenza; LPAI, low pathogenicity avian influenza; USDA, US Department of Agriculture. †There were no data on persons potentially exposed in response to detection of reassortant H5N1 virus in wild birds. We were not able to differentiate the number of responders exposed to each virus separately. ‡https://www.cdc.gov/mmwr/volumes/67/wr/mm6748a2.htm  §In response to the outbreak of infection with North American wild bird lineage H7N8 virus (LPAI that mutated to HPAI on 1 premise) in poultry in the United States during 2016. Median time a responder was on a premise was 14 d (range 1 d–44 d). ¶All 16 responders reported >1 sign or symptom: 2 had fever, 11 cough, 6 conjunctivitis, 7 sore throat, 4 rhinorrhea, 3 muscle ache, and 2 difficulty breathing. Of 11 with information, 9 (82%) were tested within 2 d of illness onset. #In response to the outbreak of infection with North American wild bird lineage H7N9 virus (LPAI with 1 mutation to HPAI) in poultry in the United States during 2017. Median time a responder was on a premise was 5 d (range 2 d–41 d). **One responder infected with coronavirus reported coughing and sneezing. One responder infected with influenza B virus reported having a fever. No other signs or symptoms were reported from responders.

These results complement data previously published for outbreaks in the United States during 2014–2015, which found no avian influenza infections in 164 persons mostly exposed while not wearing PPE (*5*). Animal model data also support these epidemiologic data. The gs/GD/96 H5N8 and reassortant HPAI H5N2 viruses can replicate efficiently in the respiratory tract of ferrets, but illness was mild and did not transmit through direct contact between ferrets (*6*). North American lineage H7N8 virus also replicated in ferrets, and pathogenicity was greater for HPAI viruses than for LPAI viruses. Limited transmission in ferrets through direct contact was observed only for the LPAI virus (*7*). Similarly, North American lineage LPAI H7N9 viruses demonstrated limited transmissibility through direct contact in ferrets (*8*). The viruses from each of these events were evaluated by using the Influenza Risk Assessment Tool (*9*), which is used to assess the potential pandemic risk. The North American H7N8 and H7N9 viruses had low risk, and the gs/GD H5N8 and reassortant H5N2 and H5N1 viruses had low to moderate risk.

## Conclusions

On the basis of these data, CDC and USDA revised the recommendations for monitoring illness in responders (https://www.cdc.gov/flu/avianflu/h5/infected-birds-exposure.htm). When responding to H5 or H7 viruses similar to those previously encountered in the United States to date, we recommend passive monitoring (i.e., self-report if ill) for persons wearing adequate PPE. For response personnel with inadequate or no PPE, or after experiencing a breach of PPE, we continue to recommend active monitoring. If personnel are responding to an avian influenza virus of unknown origin, we recommend active monitoring during exposure and for 10 days postexposure, regardless of PPE use. The change in procedure will substantially reduce the workload for safety officers and public health officials, and shift much of the reporting responsibility to the individual.

A limitation to this report is the lack of information regarding breaches in PPE. It is likely that breaches occurred, which, in turn, might have increased the risk for transmission. PPE noncompliance had been well-documented in the healthcare setting (*10*). The fact that we were unable to detect such a transmission event while actively monitoring, combined with what we know about the genetic and receptor binding characteristics of these viruses and the animal studies (*7*–*9*,*11*), might suggest that the viruses are not well adapted to humans. Another limitation was the challenge to follow-up responders after they returned to their home states. These data further support the rationale for revising the current monitoring recommendations. In contrast, if infection causes mild illness, it might have been missed. It is possible that in revising the monitoring recommendations we will risk missing a human infection. However, we might expect to detect an illness severe enough that the person seeks medical care and is tested for influenza.

The gs/GD/96 lineage clade 2.3.4.4 HPAI H5 viruses continue to cause outbreaks in poultry and wild birds in other parts of the world (*12*). One report from Canada monitored 50 household members or animal caretakers on affected farms potentially exposed to gs/GD/96 reassortant H5N2 or H5N1 viruses during 2014–2015, and no infections were identified (*13*). A recent study of 23 countries in Europe on outbreaks of infection with gs/GD/96 H5N8 virus in poultry during 2016–2017 reported no infections among 524 exposed persons who were monitored by a mixture of active and passive monitoring (*14*). Globally, many more persons have likely been exposed, but no human infections with gs/GD/96 H5N8 clade 2.3.4.4 viruses have been reported. Novel influenza virus infections in persons are reportable to the World Health Organization through the International Health Regulations (*15*). Given the nature of influenza viruses, we will continue to monitor the epidemiology and the viruses. Going forward, CDC and USDA should prospectively collect data on exposure and PPE use to better define the risk for responders exposed to avian influenza viruses.

## References

[1] US Department of Agriculture. 2014–2015 HPAI outbreak; 2016 [cited 2018 Mar 30]. https://www.aphis.usda.gov/aphis/ourfocus/animalhealth/animal-disease-information/avian-influenza-disease/defend-the-flock/2014-2015-hpai-outbreak

[2] World Health Organization. Cumulative number of confirmed human cases for avian influenza A(H5N1) reported to WHO, 2003–2018; 2018 [cited 2018 Mar 30]. http://www.who.int/influenza/human_animal_interface/2018_03_02_tableH5N1.pdf?ua=1

[3] Abdel-Ghafar AN, Chotpitayasunondh T, Gao Z, Hayden FG, Nguyen DH, de Jong MD, et al.; Writing Committee of the Second World Health Organization Consultation on Clinical Aspects of Human Infection with Avian Influenza A (H5N1) Virus. Update on avian influenza A (H5N1) virus infection in humans. N Engl J Med. 2008;358:261–73. 10.1056/NEJMra0707279 18199865

[4] US Department of Agriculture. Highly pathogenic avian influenza standard operating procedures: 8. health and safety and personal protective equipment; 2014 [cited 2019 Jan 28]. https://www.aphis.usda.gov/animal_health/emergency_management/downloads/sop/sop_hpai_health_safety.pdf

[5] Arriola CS, Nelson DI, Deliberto TJ, Blanton L, Kniss K, Levine MZ, et al.; H5 Investigation Group. H5 Investigation Group. Infection risk for persons exposed to highly pathogenic avian influenza A H5 virus–infected birds, United States, December 2014–March 2015. Emerg Infect Dis. 2015;21:2135–40. 10.3201/eid2112.150904 26583382 PMC4672413

[6] Pulit-Penaloza JA, Sun X, Creager HM, Zeng H, Belser JA, Maines TR, et al. Pathogenesis and transmission of novel highly pathogenic avian influenza H5N2 and H5N8 viruses in ferrets and mice. J Virol. 2015;89:10286–93. 10.1128/JVI.01438-15 26223637 PMC4580194

[7] Sun X, Belser JA, Pulit-Penaloza JA, Zeng H, Lewis A, Shieh WJ, et al. Pathogenesis and transmission assessments of two H7N8 influenza A viruses recently isolated from turkey farms in Indiana using mouse and ferret models. J Virol. 2016;90:10936–44. 10.1128/JVI.01646-16 27681133 PMC5110157

[8] Belser JA, Brock N, Sun X, Jones J, Zanders N, Hodges E, et al. Mammalian pathogenesis and transmission of avian influenza A(H7N9) viruses, Tennessee, USA, 2017. Emerg Infect Dis. 2018;24:149–52. 10.3201/eid2401.171574 29260672 PMC5749443

[9] Centers for Disease Control and Prevention. Summary of influenza risk assessment tool (IRAT) results; 2018 [cited 2018 Mar 30]. https://www.cdc.gov/flu/pandemic-resources/monitoring/irat-virus-summaries.htm

[10] Hinkin J, Gammon J, Cutter J. Review of personal protection equipment used in practice. Br J Community Nurs. 2008;13:14–9. 10.12968/bjcn.2008.13.1.27978 18399366

[11] Kaplan BS, Russier M, Jeevan T, Marathe B, Govorkova EA, Russell CJ, et al. Novel highly pathogenic avian A(H5N2) and A(H5N8) influenza viruses of clade 2.3.4.4 from North America have limited capacity for replication and transmission in mammals. mSphere. 2016;1:pii:e00003–16. 10.1128/mSphere.00003-16 PMC4894690 27303732

[12] Bodewes R, Kuiken T. Changing role of wild birds in the epidemiology of avian influenza A viruses. Adv Virus Res. 2018;100:279–307. 10.1016/bs.aivir.2017.10.007 29551140

[13] Murti M, Skowronski D, Lem M, Fung C, Klar S, Bigham M, et al. Public health response to outbreaks of Avian Influenza A(H5N2) and (H5N1) among poultry - British Columbia, December 2014-February 2015. Can Commun Dis Rep. 2015;41:69–72. 10.14745/ccdr.v41i04a01 29769935 PMC5937059

[14] Adlhoch C, Dabrera G, Penttinen P, Pebody R; Country Experts. Country Experts. Protective measures for humans against avian influenza A(H5N8) outbreaks in 22 European Union/European Economic Area countries and Israel, 2016–17. Emerg Infect Dis. 2018;24:1–8. 10.3201/eid2410.180269 29989531 PMC6154149

[15] World Health Organization. International health regulations (2005). [cited 2019 Jan 28]. http://www.who.int/ihr/publications/9789241580496/en/

